# Risk of suicide among U.S. veterans who deployed as part of Operation Enduring Freedom, Operation Iraqi Freedom, and Operation New Dawn

**DOI:** 10.1186/s40621-021-00332-y

**Published:** 2021-06-16

**Authors:** Tim Bullman, Aaron Schneiderman

**Affiliations:** Post Deployment Health Services, US Department of Veterans Affairs, 810 Vermont Ave, NW, Washington, DC 20420 USA

**Keywords:** Veterans, Suicide, Risk, Firearms

## Abstract

**Background:**

There has been concern about the risk of suicide among veterans returning from deployment to Afghanistan and Iraq as part of Operation Enduring Freedom, Operation Iraqi Freedom, and Operation New Dawn (OEF/OIF/OND). This study assessed suicide risk among OEF/OIF/OND veterans by gender and unit component. Firearm related suicide was also briefly examined.

**Findings:**

The study cohort was identified from records of the US Department of Defense. Vital status and cause of death through 2016 was obtained from the Mortality Data Repository, which obtains data from the National Death Index. Suicide risk was first assessed using standardized mortality ratios (SMR)s, comparing the rate of suicide among all veterans, both collectively and separately by gender and unit component (active vs. reserve/National Guard) to the expected based on the US population adjusted for age, race, sex, and calendar year. Risk of suicide among active duty compared to reserve/National Guard veterans and male compared to female veterans was assessed with hazard ratios (HR) s, generated by Cox proportional hazards models, that included the covariates race, age, marital status, rank, and branch of service. There was an increased risk of suicide when all OEF/OIF/OND Veterans were compared to the US population, (SMR = 1.42; 95%, C.I., 1.38,1.46). Both male and female veterans had an increased risk of suicide when compared to their gender specific non-veteran counterparts, (SMR = 1.40; 95%, C.I., 1.36,1.45 and SMR = 1.85; 95%, C.I., 1.60,2.13), respectively. Active duty veterans had an increased risk of suicide compared to reserve/National Guard veterans, (HR = 1.22; 95%, C.I., 1.14,1.30). Male veterans had an almost 3-fold increased risk compared to female veterans, (HR = 2.85; 95%, C.I., 2.47,3.29). Among all veteran suicides 68.3% involved a firearm, including 68.7% among males and 59.5% among females.

**Conclusions:**

All OEF/OIF/OND veterans have an increased risk of suicide compared to non-veterans. Veterans will benefit from enhanced access to mental health services and initiatives to promote suicide prevention. Strategies that emphasize lethal means safety, an evidence based suicide prevention strategy which includes increasing safe storage practices (i.e., storing firearms unloaded and locked) can help address this increased risk of veteran suicide.

## Background

Following a 2007 CBS news report about high rates of suicide among veterans (Keteyian, 2007), various legislative initiatives were passed that provided a framework for Department of Veterans Affairs (VA) efforts to prevent/reduce veteran suicides (Bullman and Kang, 2013). Included in these efforts, was an attempt to identify the extent of veteran suicides among those deployed as part of Operation Enduring Freedom, Operation Iraqi Freedom, and Operation New Dawn (OEF/OIF/OND) and among all veterans in general. In 2012 the VA released a report enumerating veteran suicides, included among the findings: there were 18 to 22 veteran suicides a day between 1999 and 2010 (US Department of Veterans Affairs, 2012). A 2018 VA report that examined veteran suicide rates over time, reported that annual suicide rates for both veterans and non-veterans increased between 2005 and 2016, with rates among veterans remaining higher than those of non-veterans (US Department of Veterans Affairs, 2018). While many recent research efforts on veteran suicides are inclusive of all veteran suicides, other studies focused on veterans who deployed as part of OEF/OIF. One study reported a suicide rate of 27.9 per 100,000 person years at risk (PYR) among a group of former active duty OEF/OIF veterans through 2009 (Kang et al., 2015), while another study reported a suicide rate of 26.1 per 100,000 PYR among both former active and reserve/National Guard OEF/OIF veterans (Reger et al., 2015).

Results from studies assessing suicide risk associated with deployment as part of OEF/OIF have produced mixed results. One study reported that among a cohort of OEF/OIF veterans who served in active duty units and sought treatment at a VA healthcare care facility for a diagnosed mental disorder had an increased risk of suicide compared to the US population (Kang and Bullman, 2008). Another study of former active component veterans who served in the military between 2001 and 2007 found that both OEF/OIF deployed and non-deployed veterans had an increased risk of suicide when compared separately to the US general population, but that OEF/OIF deployed veterans did not have an increased risk of suicide compared to non-deployed (Kang et al., 2015). Assessing suicide risk among all military personnel, i.e. both active and reserve/National Guard components, who served between October 2001 and December 2007, another study also found no association between deployment as part of OEF/OIF and risk of suicide (Reger et al., 2015), while a study limited to active duty Army personnel did find an increased risk of suicide associated with OEF/OIF deployment (Schoenbaum et al., 2014). The finding of no association between deployment and risk of suicide when non-deployed veterans are used as a comparison group, may be related to the “healthy warrior effect”. Prior to deployment those with mental health problems believed to lessen one’s ability to perform in a “hostile” environment, may be retained in the military, but are not deployed to a combat theater, Therefore, the non-deployed veteran cohort might be at greater risk for suicide than deployed (Wilson et al., 2009; Larson et al., 2008; Bullman and Schneiderman, 2019).

In studies that included both active and reserve/National Guard component veterans, the rate of suicide was higher among active veterans than reserve/National Guard veterans (Reger et al., 2015; Bullman et al., 2018). Another comparison of interest when examining suicide risk among OEF/OIF veterans, has been the differential suicide risk by gender. As expected, male veterans, both OEF/OIF deployed and non-deployed have rates of suicide that are 2–3 times that of their female veteran counterparts (Kang et al., 2015; Reger et al., 2015). Examining suicide rates over time among all veterans (Hoffmire et al., 2015) and among a cohort of OEF/OIF veterans (Bullman et al., 2015), as expected suicide rates among male veterans remained consistently higher than those of female veterans. While male veterans continue to have a higher rate of suicide compared to female veterans, female veteran suicides have been the recent focus of popular media and research (Zarembo, 2015; US Department of Veterans Affairs, 2017; McCarten et al., 2015). Finally, method of suicide has also been assessed, particularly the use of firearms. This focus is in part due to the veterans’ familiarity with and access to firearms. As in the US population, male veterans are more likely to use firearms as a method of suicide than female veterans (McCarten et al., 2015; Centers for Disease Control and Prevention, National Center for Injury Prevention and Control, Division of Violence Prevention, 2015). While the rates of firearm related suicides have increased over time for both male and female veterans, the increase has been greater among female veterans than male veterans (McCarten et al., 2015).

This current study will update the assessment of suicide risk of all OEF/OIF/OND veterans collectively, as well as when stratified by unit component, i.e. active duty vs. reserve/National Guard, and gender. The risk of suicide among this cohort has been assessed in several earlier mortality follow-ups (Kang and Bullman, 2008; Bullman et al., 2015; Bullman et al., 2018). This study differs from the previously cited Reger study (Reger et al., 2015), by extending mortality follow-up 7 years through 2016 and increasing deployment experience in the population through 2015, adding 8 years of deployment exposure and additional cohort members. The study cohort definition also differs, the Reger study selected their cohort from all those in the military between 2001 and 2007, whereas this current cohort only includes those separated from the military between 2001 and May 2015. This study also provides greater detail relative to method of suicide, with a focus on firearm related suicides. In keeping with the greater scrutiny paid to female veteran suicides, additional analyses will focus on gender comparisons, namely a comparison of suicide rates over time between male and female veterans. Finally, in a limited fashion this study looks at mechanism of suicide, focusing on firearm related suicides.

## Methods

This study’s cohort includes 1,935,168 veterans who served in either active duty component units or reserve/National Guard units as part of either OEF/OIF/OND and ended their deployment or separated from the military through May 31, 2015. Those who died while in the military, whether in theater or elsewhere, were excluded. May 31, 2015 was chosen as it was the latest deployment data available to researchers at the time this study was initiated. All military service and demographic characteristics were obtained from the U.S. Department of Defense’s (DOD), Defense Manpower Power Data Center, which is a data repository of all US military personnel records.

Mortality data was obtained by matching all cohort veterans against data included in the VA/DOD Mortality Data Repository (MDR). The MDR contains all-cause mortality for all military service members who separated from military service since 1979. At the time this study was initiated MDR had cause of death data through 2016. Cause of death and fact of death in the MDR are obtained from the National Death Index (NDI). Suicides were all deaths identified with one of the following International Classification of Disease, 10th revision (ICD-10) codes; X60-X84, Y87.0 (World Health Organization, 1992).

### Statistical analyses

Initial analysis used crude suicide rates (CR)s to approximate suicide risk among subgroups of this study’s OEF/OIF/OND veterans. CRs per 100,000 PYR for all OEF/OIF/OND veterans collectively and then by strata of interest were compared. Beginning of follow-up for veterans who served in active duty components was the date they separated or retired from the military through the end of May 2015, while beginning of follow-up for reserve/National Guard component veterans was the end date of their last OEF/OIF/OND deployment through May 2015. End of follow-up for all veterans was the earlier of date of death or December 31, 2016.

Suicide risk was also assessed by comparing the observed number of suicides among OEF/OIF/OND veterans to the expected based on the number of suicides in the US population, adjusted for age at entry to follow-up, race, sex, and calendar year. This comparison is expressed as a standardized mortality ratio (SMR) and was generated by the National Cancer Institute software, known as SEERSTAT® (National Cancer Institute, 2019). SMRs were calculated and compared for the entire cohort of OEF/OIF/OND veterans and then separately for various subgroups. Suicide risk by demographic and military service characteristics were further assessed using Cox Proportional Hazards Models generated by SAS® PHREG procedure (SAS, 2011). The Cox Model, which incorporates time at risk, was used to calculate hazard ratios (HR) s that assessed the effect of covariates on risk of suicide among the entire cohort of OEF/OIF/OND veterans. Covariates included in the model were: age at entry to follow-up; race; gender; marital status; rank; served as a ground troop, i.e. served in either Army or Marines; and service in an active duty component unit during OEF/OIF/OND deployment. Serving as ground troops while deployed, i.e. served in Army or Marines, is used as a surrogate measure of exposure to combat trauma. If combat is a risk factor for suicide, then Army and Marines personnel who had a greater likelihood of serving in closer proximity to enemy combatants than those who served in the Air Force or Navy, might be at increased risk for suicide.

Differences in suicide rates over time between the entire cohort, active vs. reserve/National Guard veterans, and male vs. female veterans were first assessed using the SAS® LIFETEST procedure (SAS Institute, 2011). LIFETEST generated separate crude hazard rates for all cohort veterans collectively, and separately for active duty veterans, reserve/National Guard veterans, male veterans, and female veterans over time since separation/deactivation from the military. Hazard rates are estimates of the probability of occurrence of an event during a specific time interval (Lee, 1980). For this analysis, hazard rates were calculated by one-year intervals from beginning of follow-up. Due to the small number of suicides among those with 14 or more years of data after entry to follow-up, analysis of suicide risk by number of years since separation/deactivation was limited to the first 13 years of follow-up. All hazard rates were calculated per 100,000 persons at risk. Differences in temporal patterns of suicide by group were further examined using Joinpoint regression software, which evaluated the potential for non-linear trends and calculated the annual percent change (APC) in suicide rates by years since separation (Kim et al., 2000).

## Results

Table 1 has selected demographic and military service characteristics for the 1,178,815 active component veterans and 756,353 reserve/National Guard component veterans included in this study. As expected, former active component veterans were younger than their reserve/National Guard counterparts; with mean age at entry to follow-up, 30.0 years and 33.7 years, respectively. Active component veterans were more likely than reserve/National Guard veterans to be single prior to their separation/deactivation from the military, 57.8% vs. 52.7%. A higher percentage of reserve/National Guard veterans than active component veterans served in either Army or Marines (73.6% vs. 59.8%). Both active and reserve/National Guard veterans were similar relative to sex and racial composition.

**Table 1:** Selected demographic/military service characteristics for OEF/OIF/OND veterans, by active vs. reserve/National Guard component

	Active		Reserve/National Guard	
Characteristics	Frequency	Percentage	Frequency	Percentage
	(***N*** = 1,178,815)		(***N*** = 756,353)	
**Age at entry to follow-up**				
17–21	37,801	3.2	45,197	6.0
22–25	435,005	36.9	159,227	21.0
26–35	423,211	35.9	251,136	33.2
36–45	214,549	18.2	190,906	25.2
46 − +	68,249	5.8	109,887	14.6
Mean age at entry to Follow-up	30.0		33.7	
**Sex**				
Male	1,038,380	88.1	664,987	87.9
Female	140,435	11.9	91,366	12.1
**Race**				
White	795,337	67.5	542,829	71.8
Non-White	383,478	32.5	213,524	28.2
**Marital Status**				
Married	497,298	42.2	357,961	47.3
Not Married	681,517	57.8	398,392	52.7
**Branch of Service**				
Ground (Army/Marines)	705,360	59.8	556,339	73.6
Non-Ground (Navy/Air Force/Coast Guard)	473,455	40.2	200,014	26.4
**Rank**				
Enlisted	1,066,263	90.5	652,264	86.2
Officer/Warrant Officer	112,552	9.5	104,089	13.8

Note. OEF/OIF/OND refers to veterans of Operation Enduring Freedom, Operation Iraqi Freedom, and Operation New Dawn

Among the entire cohort there were 4618 suicides, CR = 31.87 per 100,000 PYR. Unadjusted rates per 100,000 PYR of suicide by demographic and military service characteristics are presented separately for active and reserve/National Guard veterans, (Table 2). Active duty veterans had higher rates of suicide compared to reserve/National Guard veterans, 35.1 vs. 27.0. Male veterans, both active duty and reserve/National Guard, had higher suicide rates than their female counterparts, 38.3 vs. 11.1 and 29.1 vs. 11.3, respectively. Among both active duty and reserve/National Guard veterans, whites had higher rates of suicide than their respective non-white counterparts, 39.1 vs. 23.1 and 30.1 vs. 15.7. Rates of suicide decreased as age at entry to follow-up increased for both active and reserve/National Guard veterans, with the highest rates reported for those age 17–21, 54.5 and 50.3, respectively. Enlisted had higher rates of suicide compared to officers and those who served in the Army or Marines had higher rates of suicide than those who served in the Navy or Air Force.

**Table 2:** Crude rates of suicide by demographic and military service characteristics for OEF/OIF/OND veterans (rates and 95% confidence intervals)

	Active			Reserve/National Guard		
Characteristics	Suicides	Crude Rate^**1**^ (95% C.I.)^**2**^		Suicides	Crude Rate^**1**^ (95% C.I.)^**2**^	
**All**	2997	35.1	(33.8, 36.3)	1621	27.0	(25.7, 28.3)
**Age Group**^**3**^						
17–21	190	54.5	(47.0, 62.8)	184	50.3	(43.3, 58.1)
22–25	1403	40.8	(38.7, 43.0)	413	33.0	(29.9, 36.3)
26–35	1033	35.8	(33.7, 38.1)	536	27.9	(25.6, 30.3)
36–45	319	22.1	(19.7, 24.7)	369	23.3	(21.0, 25.9)
46 − +	52	12.0	(9.0, 15.8)	119	13.4	(11.1, 16.1)
**Race**						
White	2498	39.1	(37.6, 40.7)	1417	30.1	(28.5, 31.7)
Non-White	499	23.1	(21.1, 25.2)	204	15.7	(13.6, 18.0)
**Sex**						
Male	2884	38.3	(37.0, 39.7)	1539	29.1	(27.7, 30.6)
Female	113	11.1	(9.1, 13.3)	82	11.3	(9.0, 14.0)
**Marital Status**						
Married	996	28.1	(26.4, 29.9)	662	22.4	(20.8, 24.2)
Not Married	2001	40.0	(38.3, 41.8)	959	31.4	(29.4, 33.4)
**Rank**						
Enlisted	2891	37.4	(36.1, 38.8)	1505	28.9	(27.4, 30.4)
Officer/Warrant Officer	106	12.9	(10.6, 15.6)	116	14.7	(12.1, 17.6)
**Ground Troops**						
Yes (Army/Marines)	2088	41.7	(40.0, 43.6)	1317	29.7	(28.1, 31.3)
No (Navy/Air Force)	909	25.6	(24.0, 27.4)	304	19.4	(17.3, 21.7)

Note. OEF/OIF/OND refers to veterans of Operation Enduring Freedom, Operation Iraqi Freedom, and Operation New Dawn. Age at entry to follow-up

^1^ Crude rates are per 100,000 Person Years at Risk

^2^ 95% Confidence Interval (C.I.)

^3^ Age at entry to follow-up

Table 3 compares the rates of suicide of all OEF/OIF/OND veterans collectively and for all veterans when stratified by selected covariates to the expected based on the US population using SMRs. The risk of suicide by demographic and military service characteristics are also assessed among all OEF/OIF/OND veterans using Cox models. All veterans had an increased risk for suicide compared to the US population (SMR = 1.42; 95%, C.I., 1.38,1.46). Both active and reserve/National Guard veterans had increased risks of suicide when compared separately to the US population, (SMR = 1.60; 95%, C.I., 1.54,1.65) and (SMR = 1.18; 95%, C.I., 1.12,1.24), respectively. Examining suicide risk by gender, male veterans had an 40% excess compared to the male US population, (SMR = 1.40, 95%, C.I., 1.36,1.45), while female veterans had a 85% excess, (SMR = 1.85, 95%, C.I., 1.60,2.13), compared to female non-veterans. Both white and non-white veterans also had increased risks of suicide compared to their white and non-white non-veteran counterparts. Among the military service characteristics assessed, only veterans who were officers did not have an increased risk of suicide compared to US population, (SMR = 0.55; 95%, C.I., 0.48,0.62).

**Table 3:** Risk of suicide among OEF/OIF/OND veterans by unit component and gender

Characteristics	SMR^**1**^ (95% C.I.)^2^		HR^**3**^	(95% C.I.)^**2**^
**All OEF/OIF**	1.42 (1.38, 1.46)			
**Unit Component**				
Active	1.60	(1.54,1.65)	1.22	(1.14,1.30)
Reserve/National Guard	1.18	(1.12,1.24)	Referent	
**Age at Entry to Follow-up**^**4**^			0.98	(0.97,0.98)
**Race** (All veterans)				
White	1.39	(1.35,1.44)	1.72	(1.59,1.86)
Non-White	1.77	(1.64,1.91)	Referent	
**Marital Status** (All veterans)				
Not Married			1.12	(1.05,1.20)
Married			Referent	
**Rank** (All veterans)				
Enlisted	1.57	(1.52,1.61)	1.94	(1.69,2.23)
Officer	0.55	(0.48,0.62)	Referent	
**Ground Troop** (All veterans)				
Yes	1.64	(1.59,1.70)	1.39	(1.30,1.49)
No	1.07	(1.01,1.13)	Referent	
**Sex** (All veterans)				
Male	1.40	(1.36,1.45)	2.85	(2.47,3.29)
Female	1.85	(1.60,2.13)	Referent	

Note. OEF/OIF/OND refers to veterans of Operation Enduring Freedom, Operation Iraqi Freedom,

and Operation New Dawn

^1^ Standardized Mortality Ratio (SMR). Ratio of observed to expected based on US population, all adjusted for age and calendar year. Other adjustments depend on veteran subgroup

^2^ 95% Confidence Interval (C.I.)

^3^ HR = Hazard Ratio of suicide derived from Cox Model that included the covariates, age, race, sex, branch of service, and unit component

^4^ Age is included as a continuous variable in Cox Model

Assessing suicide risk among the entire cohort of OEF/OIF/OND veterans by demographic and military service characteristics included in a Cox Proportional Hazards model, males had an almost 3-fold increased risk of suicide compared to females (HR = 2.85; 95%, C.I., 2.47,3.29) and active duty veterans had an increased risk of suicide compared to reserve/National Guard veterans (HR = 1.22; 95%, C.I., 1.14,1.30). Whites had an increased risk of suicide compared to non-whites (HR = 1.72; 95%, C.I., 1.59,1.86) and not married had an increased suicide risk compared to married (HR = 1.12; 95%, C.I., 1.05,1.20). Enlisted veterans had an almost 2-fold increased risk of suicide compared to officers, (HR = 1.94; 95%, C.I., 1.69,2.23), and ground troops, i.e. served in Army or Marines, had an increased risk of suicide compared to non-ground troops, (HR = 1.39; 95%, C.I., 1.30,1.49).

**Fig. 1:**
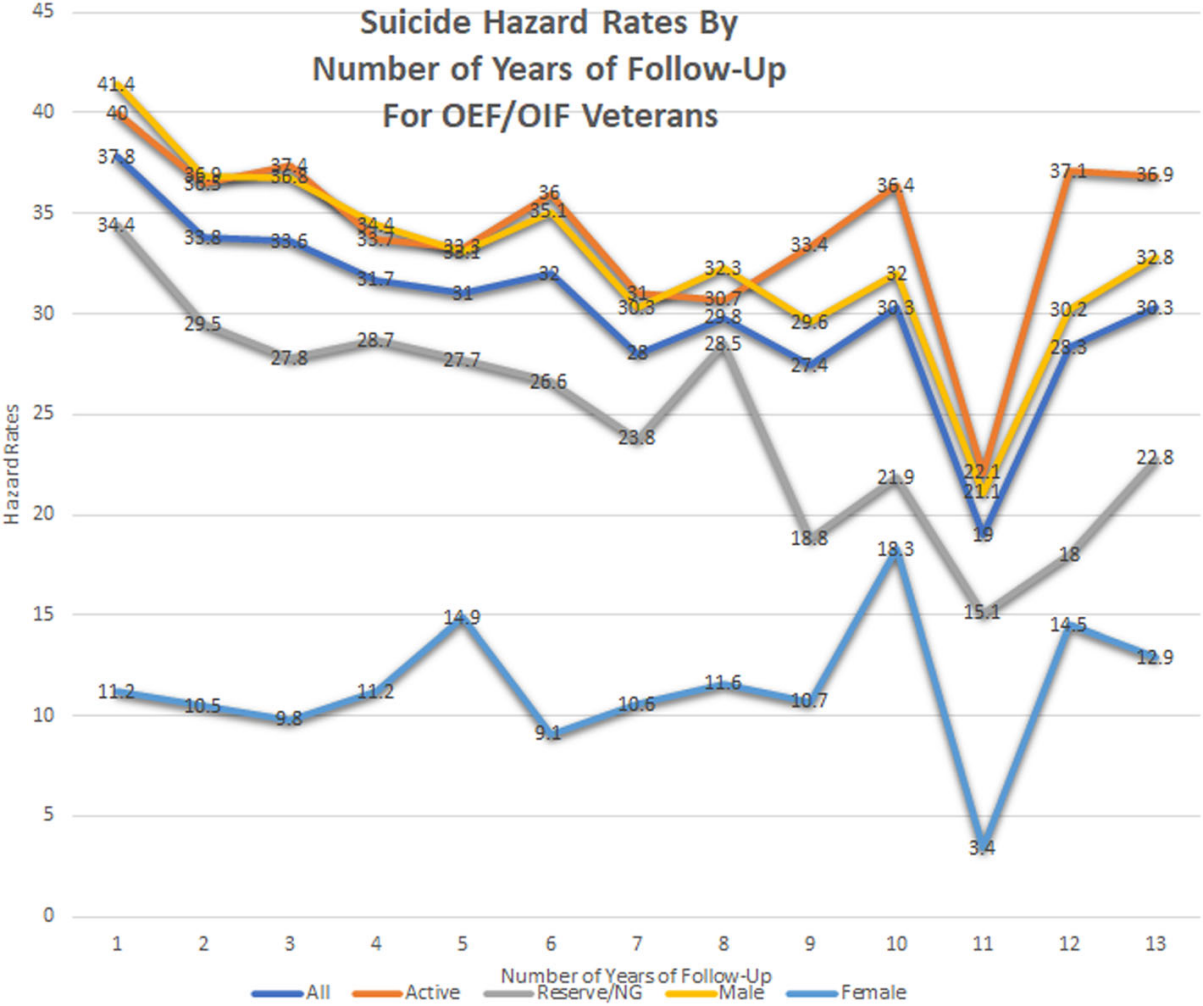
Suicide hazard rates by number of years of follow-up for OEF/OIF/OND veterans

Figure 1, Suicide Hazard Rates by Number of Years of Follow-up for OEF/OIF/OND Veterans, plots the crude hazard rates of suicide for all OEF/OIF/OND veterans collectively, separately by unit component, i.e. active vs. reserve/National Guard, and by gender. All hazard rates are per 100,000 persons at risk and are plotted by number of years since entry to follow-up, i.e. date of separation for active component veterans and end date of last OEF/OIF/OND deployment for reserve/National Guard veterans. The highest suicide rate, as approximated by hazard rate, for all OEF/OIF/OND collectively and when stratified by unit component occurred in the first year of follow-up, hazard rate = 37.8 for all OEF/OIF/OND veterans, hazard rate = 34.4 for reserve/National Guard veterans, and hazard rate = 40.0 for active duty veterans. While the suicide rates for all three groups showed variation over 13 years of follow-up, the APC in rates of suicide for all OEF/OIF/OND collectively, for active duty OEF/OIF/OND veterans, and for reserve/National Guard OEF/OIF/OND veterans indicated a statistically significant decrease in risk of suicide over time for all three groups. The APC for all OEF/OIF/OND veterans was (^−^ 3.0; 95% C.I., ^−^ 4.4,^−^ 1.6); for active duty veterans

(APC = ^**−**^ 1.9; 95%, C.I., ^−^ 3.6,^−^ 0.2); and for reserve/National Guard veterans (APC = ^−^ 4.7; 95%, C.I., ^−^ 6.5,^−^ 2.8). Male OEF/OIF/OND veterans, similar to the total cohort of veterans, had their largest risk of suicide after one year of follow-up, HR = 41.4, whereas female OEF/OIF/OND veterans had their largest suicide risk after ten years of follow-up, HR = 18.3. Like the other subgroups examined, males had a statistically significant trend of decreased risk of suicide over 13 years, (APC = ^−^ 3.2; 95%, C.I., ^−^ 4.6,^−^ 1.9), whereas female veterans have a trend of increased risk of suicide over time, although it was not statistically significant, (APC = 2.0; 95%, C.I. ^−^ 2.5,6.6).

The extent of suicides involving firearms was also examined. Among all suicides 68.3% involved use of a firearm. A higher percentage of male suicides involved a firearm then did female suicides, 68.7% vs. 59.5%. After firearm related suicides, the most frequently utilized methods of suicide included; strangulation, i.e. hanging, 19%; self-poisoning using drugs, 5.4%; and self-poisoning with gas, 2.2%.

## Discussion

This study assessed suicide risk through the end of 2016 among veterans deployed as part of OEF/OIF/OND. Suicide risk was examined for all OEF/OIF/OND veterans collectively, and separately by gender and unit component, i.e. active vs. reserve/National Guard. Assessing risk of suicide among all OEF/OIF/OND veterans relative to US population, OEF/OIF/OND veterans had a 42% excess of suicide. Among all OEF/OIF/OND veterans the following had an increased risk for suicide; active duty compared to reserve/National Guard; whites compared to non-whites; not married compared to married; enlisted compared to officers; ground troops compared to non-ground troops; and males compared to females.

Rates of suicide for all OEF/OIF/OND veterans collectively, and when examined separately for male, active, and reserve/National Guard veterans had a trend of statistically significant decrease over time since the veteran entered follow-up. (Fig. 1) The rates of suicide for female veterans had a pattern of increase, although not statistically significant, over the 13 years of follow-up.

Earlier mortality follow-ups of the OEF/OIF/OND cohort also reported larger rates of suicide among active duty compared to reserve/National Guard veterans (Kang and Bullman, 2008; Bullman et al., 2018). As cited here and expanded on in a previous study of this cohort, the increased risk of suicide among active duty relative to reserve/National Guard veterans may be related to greater social integration among reserve/National Guard veterans compared to active duty veterans (Bullman et al., 2018). Research has reported that higher levels of social integration are associated with lower risk of suicide (Duberstein et al., 2004; Eng et al., 2002).

In general, the higher risk of suicide among veterans compared to the US population, may be related to availability of firearms. A study comparing firearm ownership among veterans to non-veterans, reported that veterans were more than twice as likely to own a firearm as non-veterans, 44.9% vs. 20% (Cleveland et al., 2017). The greater frequency of firearm ownership among veterans than non-veterans is also reflected in the findings from a 2018 VA report on suicide (US Department of Veterans Affairs, 2018). Reviewing mortality data for veterans and non-veterans between 2005 and 2016, a higher percentage of veteran suicide in 2016 involved the use of a firearm than non-veteran suicides, 69.4% vs. 48.4%, respectively.

Among this study’s limitations was the potential for misclassification and underreporting of suicide. It is not known whether there would be differential reporting of suicide for former military personnel relative to that reported among the US general population. If the underreporting of suicide was greater among former military than the US general population, the risk of suicide among veterans would be even larger than that reported in this study. Because this study did not have a non-deployed veteran comparison group, it is not possible to discern if deployment as part of OEF/OIF/OND specifically, or service in the military in general, is a risk factor for suicide. However, recent studies of both deployed and non-deployed OEF/OIF veterans reported that military service in general, and not OEF/OIF deployment specifically, was associated with an increased risk of suicide (Kang et al., 2015; Reger et al., 2015). Even if a non-deployed veteran group had been available, the reported ‘healthy warrior effect’, where those with perceived mental health problems are not likely to be deployed to a combat theater, could artificially diminish suicide risk in a deployed group (Wilson et al., 2009; Larson et al., 2008; Bullman and Schneiderman, 2019). Another inherent limitation for this study and other studies of OEF/OIF/OND veterans is the inability to assess exposure to combat trauma. OEF/OIF/OND deployments can include service in countries other than Iraq and Afghanistan, which may have been less likely to have exposed participants to combat. This study also lacked any data on levels of combat and trauma experienced by OEF/OIF/OND veterans while deployed, as well as number and length of deployments.

After 15 years of follow-up OEF/OIF/OND veterans continue to have a suicide rate that exceeds that of the US population. The greater reliance on firearms as a method suicide among veterans relative to non-veterans (US Department of Veterans Affairs, 2018) may in part explain their higher rate of suicide when compared to US population, i.e. firearms as a method of suicide is more likely to result in death than other methods.

That 68% of this study’s suicides involved a firearm, clearly signals the need to address the ownership of firearms by vulnerable groups of veterans. Suicide prevention efforts might benefit by focusing on firearm access/ownership by veterans who are in mental distress. A recent effort to address the issue of firearm ownership and suicide among veterans was included in the 2020 “Commander John Scott Hannon Veterans Mental Health Care Improvement Act”. As originally written the bill included support for a suicide prevention strategy known as ‘lethal means safety’, where health care workers who treat veterans would be trained on how to talk with at-risk patients about the danger of having guns in the house and how to reduce that risk. However, this provision of the legislation was not included in the final approved bill. Other legislation that neither focused on veterans or firearm related suicide, banned the sale or providing a firearm to any individual who has been adjudicated as mentally ill (18 U.S.C. § 922 G(4)). The extent to which veterans who died from a firearm related suicide would have met the characterization as provided for in the law is unknown. Identifying at risk veterans prior to their purchase of a weapon, may be precluded by patient confidentiality laws. In addition, use of medical records for screening gun sales or issuing a gun license, may discourage veterans from seeking medical care for their mental health concerns. The extent to which using medical records might be used to screen for potential firearm related suicides was assessed in a study that linked suicide data with hospitalization data in the state of Utah. This study, which did not include veteran status, reported that 25% of firearm suicides had been seen in a hospital with a behavioral health diagnosis in the year before their death and only 6% were seen for a suicide attempt or self-harm (Barber et al., 2019).

While its effectiveness has yet to be assessed by this or any other study, an innovative and potentially more acceptable approach to veteran suicide prevention was initiated by the group Cover Me Veterans (Cover Me Veterans, 2021). Through the Cover Me Veterans program, veterans can receive free of charge a kit to create a vinyl wrap, or skin, to be placed on his/her gun, that has a photo or image of something that has an emotional connection to the veteran, such as spouse, child or parent. The rationale being that looking at an emotionally evocative image at the moment immediately preceding the act of suicide might encourage self-reflection on the part of the veteran.

## Conclusions

This study, as has been reported in previous follow-ups of this cohort of OEF/OIF/OND veterans, found that these veterans remain at increased risk for suicide compared to the US population. As in the US population, the most common method of suicide involves the use of firearms. In addition to continuing to enhance access to mental health services for veterans, future efforts to reduce suicide among veterans should continue to emphasize lethal means safety, an evidence based suicide prevention strategy which includes, among other things, increasing safe storage practices (i.e., storing firearms unloaded and locked).

## Data Availability

Data are not available because of data use agreement restrictions.

## Abbreviations

VA: Department of veterans affairs
OEF/OIF/OND: Operation enduring freedom, operation iraqi freedom, operation new dawn
PYR: Person years at risk
DOD: Department of defense
MDR: Mortality data repository
NDI: National death index
ICD: International classification of disease
CR: Crude rate
SMR: Standardized mortality ratio
HR: Hazard ratio
C.I.: Confidence interval
APC: Annual percentage change
